# Multiple magma reservoirs for the 1707 eruption of Fuji volcano, Japan

**Published:** 2004-02-01

**Authors:** Mitsuhiro Yoshimoto, Toshitsugu Fujii, Takayuki Kaneko, Atsushi Yasuda, Setsuya Nakada

**Affiliations:** Earthquake Research Institute, University of Tokyo, 1-1-1, Yayoi, Bunkyo-ku, Tokyo 113-0032

**Keywords:** Magma mixing, multiple magma chambers, basaltic subplinian, Fuji volcano

## Abstract

In 1707, approximately 0.7 km^3^ of magma erupted from new vents on the southeastern slope of Fuji volcano. The air-fall clasts of this explosive eruption are composed of vesicular dacite pumice, andesitic dense scoria, basaltic dense scoria, and vesiculated basalt scoria, stratified from bottom to the top of the fallout unit. Some compositional gaps are found in the variation from basalt to dacite indicating that three independent magmas, basalt, andesite and dacite, existed just prior to the eruption. Andesite and dacite magmas are mixed just prior to or during the eruption showing a linear two component mixing in the major and trace element concentration. Basalt in the later stage of the eruption shows no compositional affinity with the above two magmas. Basalt magma might have acted as a heat source to remobilize the andesite and dacite magmas, and the explosive eruption of basalt could have been caused by the abrupt pressure release due to precursory mixing and eruption of dacite and andesite magmas.

## Introduction

The 1707 eruption, one of the most explosive eruptions in the eruptive history of Mt. Fuji, issued andesite and dacite pumice as well as basalt scoria (e.g. Tsuya1)). The ejecta were dispersed eastward and the thickness of the air-fall deposit exceeded four meters at a site 8 km from the vent and reached a few centimeters even at Tokyo, 100 km from the vent. The accumulated volume is estimated to have been approximately 0.7km^3^ dense rock equivalent.2) The eruptive products were initially dacitic ejecta, replaced by basaltic one on the following day. The eruption continued two weeks. Through the hundred thousand year history of Fuji volcano, only two eruptions, including the 1707 eruption, are known to have issued dacitic magma.

As the ejecta shows wide compositional variation from dacite through basalt,3)–5) existence of a compositionally zoned magma chamber prior to the 1707 eruption has been proposed (e.g. Tsukui4)). Koyaguchi5) discussed the mechanism to form the zoned magma chamber, and concluded that the andesitic composition could be formed by boundary layer convection at the interface between dacite and underplating basalt magmas.

In this paper we discuss the magma storage system of the 1707 eruption based on new analyses on air-fall clasts from the outcrops that preserve the whole sequence of the 1707 eruption.

## Eruptive products from the 1707 eruption

The 1707 air-fall deposit is divided into four major fall units; Ho-I to Ho-IV in ascending order.6) Ho-I was emplaced during the initial plinian eruption from Hoei No. 2 and 3 craters. Ho-I is composed of well-sorted and normally graded vesicular pumice, occasionally containing banded pumice and obsidian clasts as juvenile material. Ho-I is subdivided into two units. Lower Ho-1a is composed mainly of white to light brown pumice, and upper Ho-Ib contains dark brown pumice. Volumetric ratios of dark brown pumice and banded pumice to the white pumice increase toward the upper part of the unit. Ho-II, which consists of dark-gray dense scoria and rarely subangular to rounded pumice, was also formed by plinian eruption from Hoei No. 2 and 3 craters. The dense scoria in the lower part of Ho-II is angular fracture-bounded. Ho-III and IV are composed of basaltic scoria erupted from Hoei No. 1 crater located at the highest altitude among the three craters formed during the eruption. Ho-III is alternation of coarse- and fine-grained black poorly-vesiculated scoria. At some horizons, very small amounts of subangular to rounded pumice clasts are found. Ho-IV consists of black vesiculated scoria lacking pumiceous clasts.

## Multiple magmas for the 1707 eruption

Air-fall clasts from the whole sequence were carefully examined to eliminate the banded pumice and only the homogeneous clasts were selected for the whole rock chemical analysis. XRF analysis was performed on a single clast to eliminate any possible mixture of mingled magmas. Several homogenous clasts were picked up from each unit of 22 fall units at an outcrop 8 km east from the vent (Dainichi-do).

**Fig. 1:**
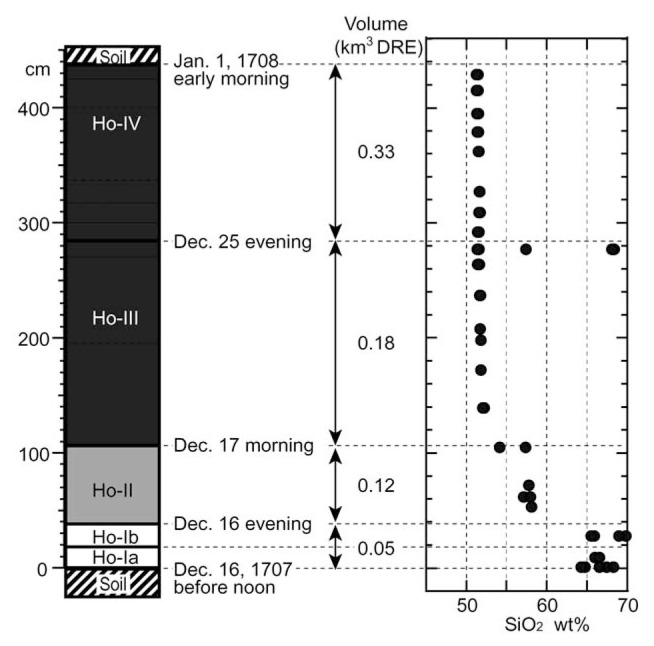
Stratigraphic variation of whole-rock SiO_2_ contents of air-fall clasts of the 1707 eruption. Chronology of the eruption and stratigraphic division follow Miyaji6) and Miyaji and Koyama.7)

**Fig. 2:**
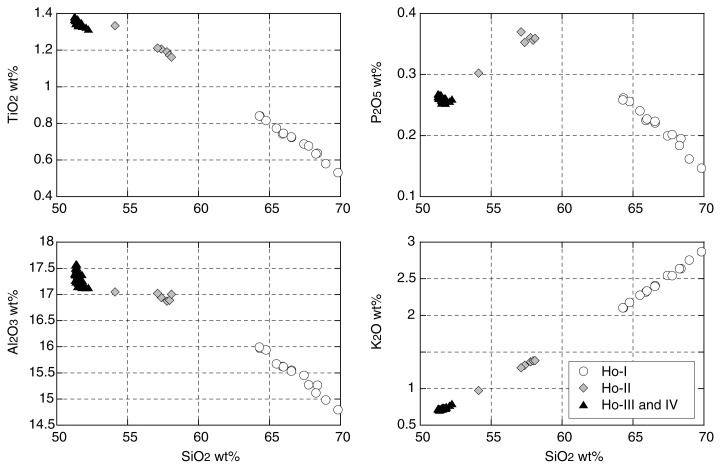
Selected variation diagrams of whole-rock major oxides of the 1707 eruption. Open circles: pumice of Ho-I; gray diamonds: dense scoria of Ho-II; solid triangles: scoria of Ho-III and IV.

SiO_2_ content of the air-fall clasts varies from 70 wt% to 51 wt% with increase in height (Fig. 1). Andesite and dacite in the upper part of Ho-III could be due to accidental entrainment of Ho-I pumice near vent. Several compositional clusters are recognized; 64.3–69.8 wt% SiO_2_ in Ho-I, 57.1–58.1 wt% SiO_2_ in Ho-II, and 51.3–52.2 wt% SiO_2_ in Ho-III and IV. Distinct compositional gaps exist between Ho-I and II and between Ho-II and III. The composition of clasts in Ho-IV is almost identical to that of Ho-III. Therefore, three different magma groups are distinguished based on SiO_2_ content: dacite (Ho-I), andesite (Ho-II), and basalt (Ho-III and IV). Andesite and dacite form a linear trend of two-component mixing. However basalt is not on the extension of the trend. These features are clearly shown in the variation of TiO_2_, Al_2_O_3_, P_2_O_5_ and K_2_O against SiO_2_ (Fig. 2). These compositional variations strongly indicate that andesite magma erupted in 1707 cannot be produced by mixing of basaltic and dacitic magmas from the same eruption.

**Fig. 3:**
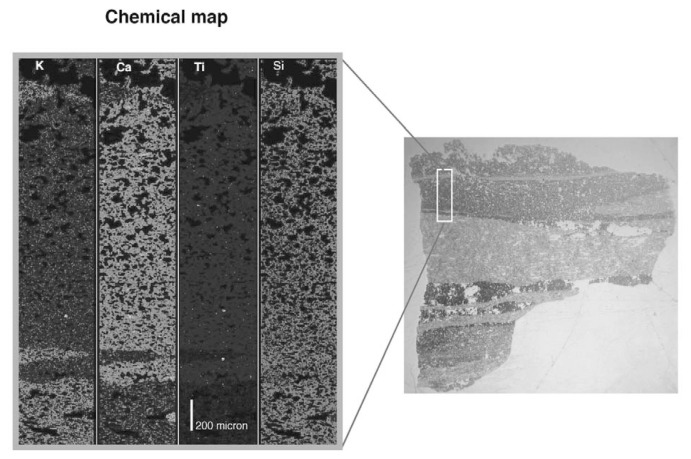
Microscopic photograph of a banded pumice from Ho-Ib (right) and chemical map of the banded part (left).

Banded pumice often found in Ho-I indicates, however, that some kind of magma mixing occurred during the eruption. Microscopic examination of banded pumice shows banding is composed of alternating dark, less vesicular bands and white vesicular bands. Thickness of the bands ranges from several tens of micrometers to several millimeters. Both dark and white bands contain many microlites and the chemical analysis of each band is not possible. Interstitial glasses in the dark and white bands contained 66 wt% and 77 wt% in SiO_2_, respectively (Table I), indicating that the dark band has an andesitic composition. No bands of basaltic composition are observed in the banded clasts. Even thin bands less than 20 micrometers show sharp boundaries and no diffusional gradation of composition is observed across the boundary (Fig. 3). These features indicate that the banded texture was quenched just after magma mixing leaving insufficient time for diffusional homogenization between andesite and dacite liquids. These lines of evidence suggest that the andesitic and dacitic magmas were derived from distinct magma chambers just prior to the eruption. The apparent linear compositional variation between andesite and dacite (Fig. 2) is probably caused by insufficient elimination of microscopically banded clasts in spite of the careful selection of analytical samples.

**Fig. 4:**
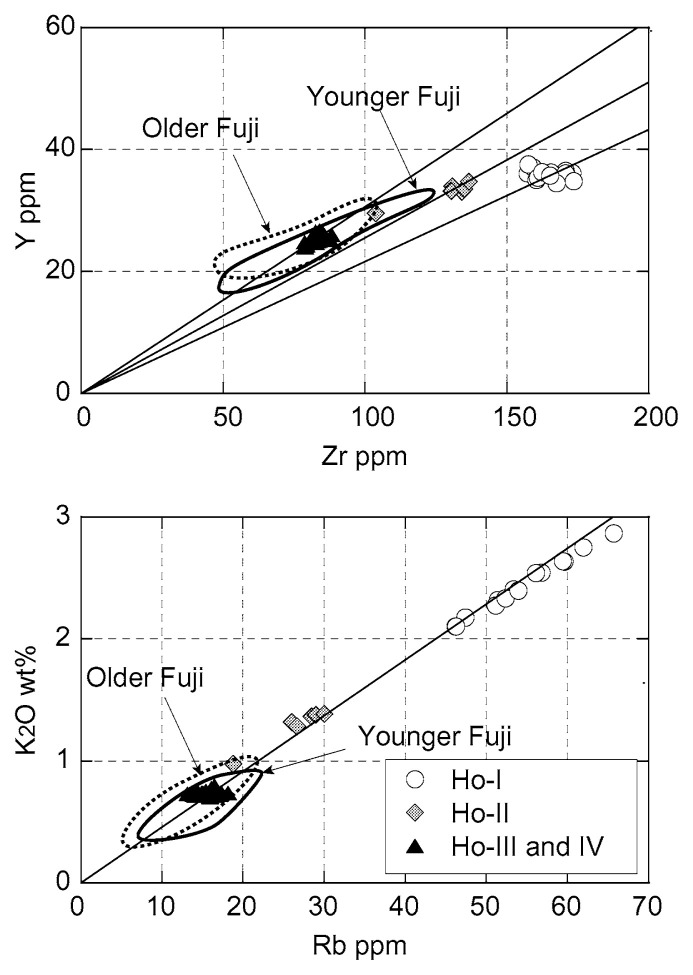
Selected incompatible-element ratios of the 1707 eruption. Symbols are the same as in Fig. 2.

The distinct inflection in the compositional variation indicates that the basalt magma issued during the 1707 eruption is not directly related to the formation of the andesite magma and must have accumulated in a different magma reservoir before the eruption. Although the andesite and dacite cannot be derived directly from the basaltic magma triggered 1707 eruption, they are not so much different from averaged basalts of Fuji volcano in terms of trace element ratios, indicating that both andesite and dacite could be formed by fractional crystallization of an older basaltic magma (Fig. 4). Slight difference in Zr/Y ratio between basalts and silicic rocks suggests contribution of pyroxene in the differentiation process.

## Scenario of the 1707 eruption

The sequence of the 1707 eruption could be explained by the following model. Before the eruption dacite and andesite magmas were stored separately in discrete magma chambers. Ascending dense basaltic magma underplated the andesite magma chamber without injection, and supplied heat to remobilize the andesite magma. The heated and remobilized andesite magma injected the dacite magma, triggering vesiculation of the dacite. Vesiculation at the bottom of the injected chamber may have resulted in vigorous convection and consequent vesiculation of both magmas. This process lead to the successive explosive eruptions of dacitic and andesitic magmas. Heterogeneous magma mixing between dacite and andesite occurred in the magma chamber and/or conduit during ascent. The explosive eruption caused an abrupt pressure release above the basaltic magma chamber. Therefore basalt that still maintained a small amount of water at some depth could vesiculate and this lead to a subplinian eruption. This mechanism satisfactorily explains the compositional gap between basalt and andesite.

## Figures and Tables

**Table I:** Major oxides in matrix glasses from the banded pumice

	White band	Dark band
SiO_2_	76.56	66.33
TiO_2_	0.37	1.26
Al_2_O_3_	13.57	13.81
FeO^*^	0.97	7.69
MnO	0.04	0.03
MgO	0.35	1.38
CaO	2.54	4.42
Na_2_O	2.19	2.26
K_2_O	3.22	2.48
P_2_O_5_	0.19	0.35
